# Equivalent Porous Media (EPM) Simulation of Groundwater Hydraulics and Contaminant Transport in Karst Aquifers

**DOI:** 10.1371/journal.pone.0138954

**Published:** 2015-09-30

**Authors:** Reza Ghasemizadeh, Xue Yu, Christoph Butscher, Ferdi Hellweger, Ingrid Padilla, Akram Alshawabkeh

**Affiliations:** 1 Department of Civil and Environmental Engineering, Northeastern University, Boston, Massachusetts 02115, United States of America; 2 Department of Engineering Geology, Institute of Applied Geosciences, Karlsruhe Institute of Technology, 76131, Karlsruhe, Germany; 3 Department of Civil Engineering and Surveying, University of Puerto Rico, Mayaguez, Puerto Rico 00682, United States of America; Tsinghua University, CHINA

## Abstract

Karst aquifers have a high degree of heterogeneity and anisotropy in their geologic and hydrogeologic properties which makes predicting their behavior difficult. This paper evaluates the application of the Equivalent Porous Media (EPM) approach to simulate groundwater hydraulics and contaminant transport in karst aquifers using an example from the North Coast limestone aquifer system in Puerto Rico. The goal is to evaluate if the EPM approach, which approximates the karst features with a conceptualized, equivalent continuous medium, is feasible for an actual project, based on available data and the study scale and purpose. Existing National Oceanic Atmospheric Administration (NOAA) data and previous hydrogeological U. S. Geological Survey (USGS) studies were used to define the model input parameters. Hydraulic conductivity and specific yield were estimated using measured groundwater heads over the study area and further calibrated against continuous water level data of three USGS observation wells. The water-table fluctuation results indicate that the model can practically reflect the steady-state groundwater hydraulics (normalized RMSE of 12.4%) and long-term variability (normalized RMSE of 3.0%) at regional and intermediate scales and can be applied to predict future water table behavior under different hydrogeological conditions. The application of the EPM approach to simulate transport is limited because it does not directly consider possible irregular conduit flow pathways. However, the results from the present study suggest that the EPM approach is capable to reproduce the spreading of a TCE plume at intermediate scales with sufficient accuracy (normalized RMSE of 8.45%) for groundwater resources management and the planning of contamination mitigation strategies.

## Introduction

Karst aquifers account for 25% of groundwater resources in the world and 40% in the US [1]. They are formed when the dissolution process in primarily soluble carbonate rocks creates complex networks of preferential flow pathways, such as solutionary fractures and conduits, within the rock matrix (karstification). Conduits are crucial for groundwater flow and contaminant transport in karst aquifers [2], but their distribution is often unknown, thus limiting the applicability and validity of the numerical models that require detailed data on conduits [3]. Subsurface flow within the aquifer ranges from laminar to turbulent, with laminar flow in the rock matrix and predominantly turbulent flow in conduits, depending on flow velocities [4]. Karst areas include swallets, sinkholes, infiltrating streams, and other highly porous surface features that limit the availability of surface water, making groundwater the primary water resource for domestic, agricultural, and industrial utilization.

Traditional simulation of groundwater hydrodynamics with numerical models based on Darcy’s law may not be directly applicable for modeling flow in karst [5, 6]. Such models are typically used for laminar groundwater flow regime and slow groundwater velocity conditions and their application in karst aquifers require extra attention. In an Equivalent Porous Media (EPM) approach for karst groundwater systems, the default assumption is that carbonate aquifers’ behavior is equivalent to porous media for both flow and transport. Also known as single continuum porous equivalent approach (SCPE), heterogeneous continuum approach, smeared conduit approach, or single continuum approach [7, 8, 9, 10], it is the simplest distributed modeling approach for karst aquifers. For the reasons outlined above, its ability to simulate groundwater flow in karst, however, is limited.

The EPM approach assumes that the rock matrix including fractures and conduit networks can be represented by an equivalent porous medium with equivalent hydraulic conductivity in a certain area [11, 12]. In highly karstified aquifers, however, the contaminant transport may depend primarily on the karst conduit network rather than matrix hydraulic conductivity. EPM models often do not distinctly account for preferential flow; instead, they approximate the overall local conductivity of the matrix as well as possible fractures and conduit networks with an enhanced equivalent conductivity [5, 13]. Despite this limitation, it can lead to representative results depending on the degree of aquifer karstification and the scale of the modeling effort. Generally, the EPM approach is more suitable for regional scales rather than local and intermediate scales [5, 14].

Scanlon et al. [5] evaluated the accuracy of two different EPM approaches, lumped parameter and distributed parameter, for simulating regional scale (330 km^2^) groundwater flow in the highly karstified Barton Springs Edwards aquifer in Austin, Texas. Both models simulated the temporal variations in spring discharge fairly accurate, but the latter approach reproduced the effect of pumping on groundwater levels, evaluated the potentiometric surface at different times, and spring discharge more accurately. According to their study, it is practical to simulate karstic aquifers as equivalent porous media for groundwater flow but not for contaminant transport (especially at local scales) or to delineate protection zones for wells or springs because transport velocities may be substantially underestimated.

Using both porous medium and conduit network simulations, Worthington [6] investigated high-permeability interconnected conduits in the limestone aquifer at Mammoth Cave (Kentucky, USA) at a regional scale (area of 258 km^2^) both experimentally and numerically to explore if the aquifer behaves more similar to a porous medium or to a karst aquifer with flow both in the rock matrix and karst conduits. While the underground drainage pattern was comparatively well-known from previous tracer studies, that study identified a significant difference in the simulated aquifer behavior with and without considering conduits; especially at local scales, the EPM approach was inadequate to simulate solute transport, represent spring hydrographs, and to interpret tracer test results.

Using MODFLOW 2000, Putnam and Long [15] developed a regional EPM flow model for the Madison and Minnelusa aquifers (area of 2590 km^2^) to synthesize available hydrogeological data and quantify the regional water budget. Saller et al. [16] further developed the above model by including high conductivity zones, representing karst conduits in the EPM model, which resulted in a better match with the observed transient well data; however, the simulated spring discharge was not improved and was similar to the original EPM.

If sufficient geologic data are available so that the location and characteristics of conduit networks within karst aquifers are fairly well known, some investigators have used MODFLOW to simulate those networks (on a scale order of less than 100 m to several kilometers) with various techniques, including lines of drain cells and internal sinks [17, 18] and lines of high hydraulic conductivity cells [19]. Although not directly comparable, results from above studies may be applicable to other karst aquifers on a case-by-case basis. Yet there remain questions as to how reliable and to what temporal resolution the EPM approach can replicate groundwater level fluctuation in karst or whether EPMs can simulate the long-term fate and transport of point source contamination.

The present study evaluates the utility of the Equivalent Porous Media (EPM) approach to simulate dynamic groundwater hydraulics and contaminant transport in highly permeable karst unconfined aquifers using an example from the North Coast limestone aquifer system in Puerto Rico at an intermediate scale (area of 132.5 km^2^ and scale order of 10 kilometers). The three dimensional finite difference model MODFLOW 2005 [20] was used for simulating flow, and the transient transport model MT3DMS [21] was used to simulate the spreading of a point source TCE contamination at a Superfund site. The main goal of this study is to evaluate if the EPM approach is suitable and practical for the assessment of major contaminated sites in karst areas, based on available data and the study’s scale and purpose.

## Materials and Methods

### Ethics Statement

No specific permits were required for the described field studies, nor for the locations and activities. The studied location is not privately-owned or protected in any way, and the field studies did not involve endangered or protected species.

### Study Site

**Fig 1:**
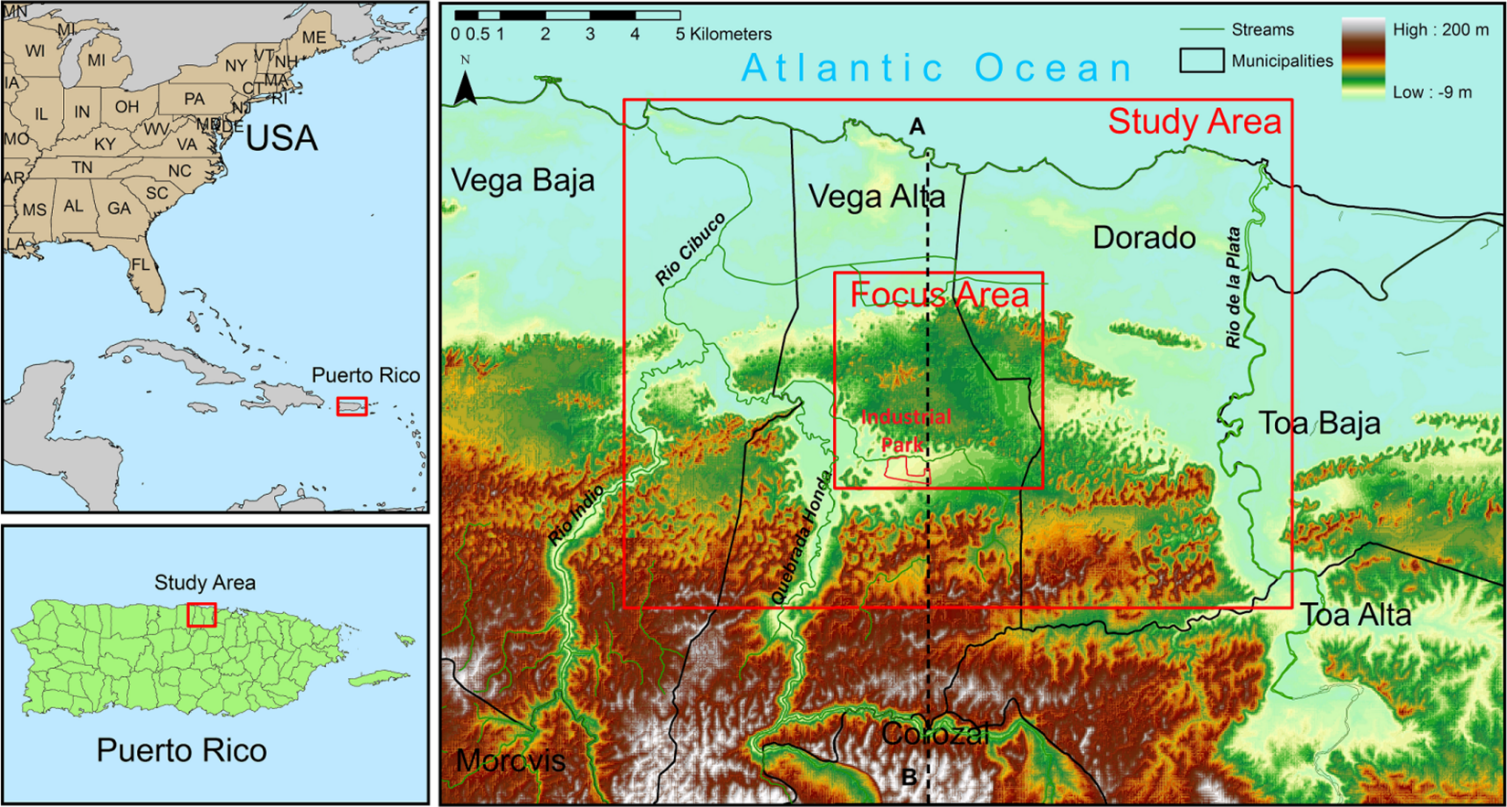
Location of study site in northern Puerto Rico. The map includes study area boundary, municipalities, major streams, focus area, Industrial Park, land surface elevation, and position of cross-section A-B.

The study was carried out at a test site within the north coast region of Puerto Rico. Karst limestone features cover 27.5% of the land surface of Puerto Rico (Fig 1). The Northern Karst Belt covers an area of approximately 2000 km^2^ (16% of the island) and elevates at heights ranging from about 300 m in the mountainous areas to sea level at the coast (Fig 1) [22]. This aquifer system is the largest aquifer of the island and contains the island’s most extensive freshwater aquifer, accounting for 80% of northern Puerto Rico’s groundwater resources [23].

#### Geology and hydrology

**Fig 2:**
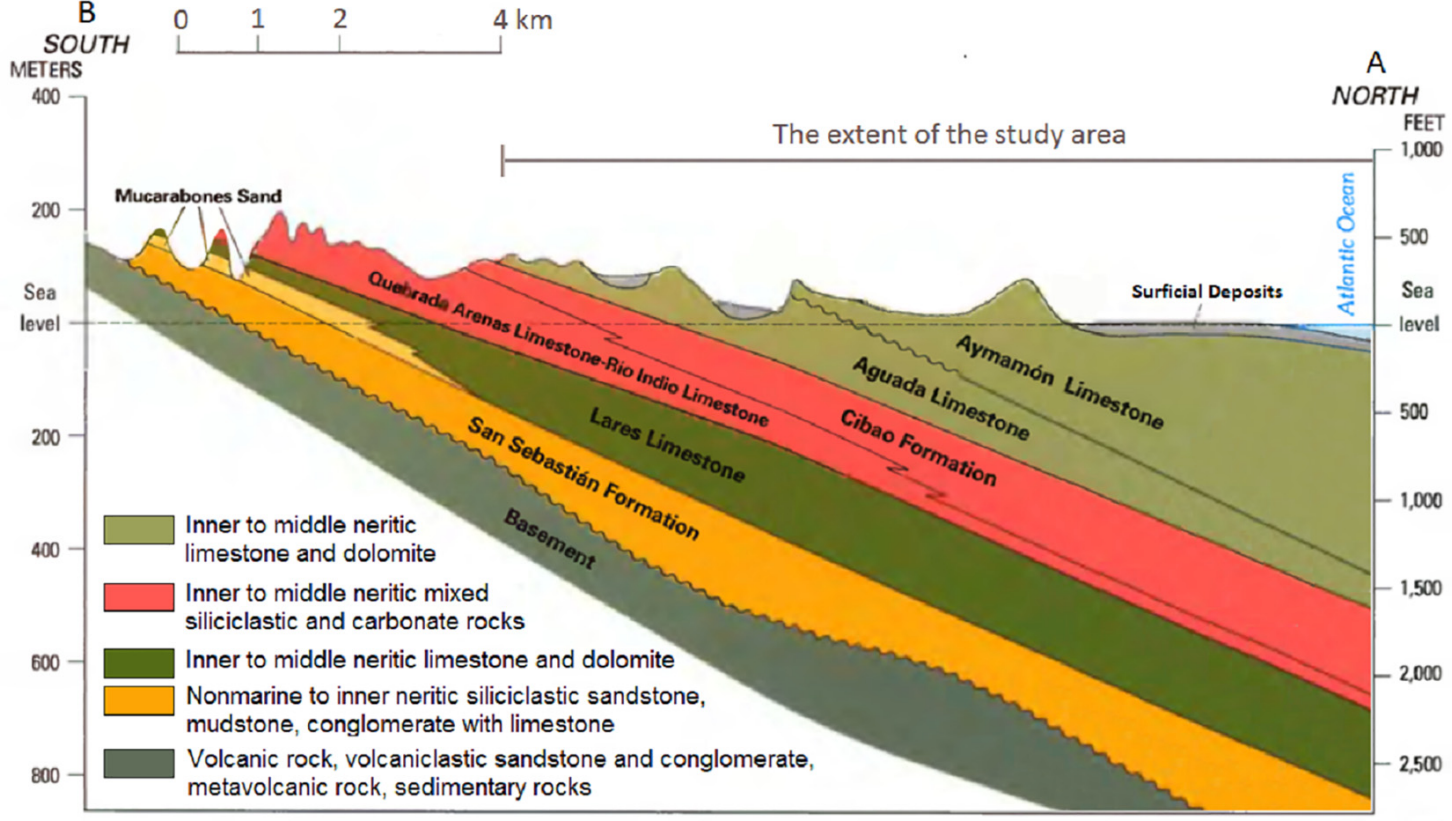
Geologic formations of the North Coast Aquifer system. The geologic sections are along cross-section A-B (see Fig 1).

The North Coast limestone aquifer system of Puerto Rico is sub-divided into four generalized hydrogeological units: the upper water-table aquifer, the middle confining unit, the lower confined aquifer, and the basal confining unit [24]. The thickness of the karst aquifer system increases from the south (Southern Karst Uplands) to the central part of the Northern Karst Belt (Karst Upland Plateau), and subsequently decreases to the coastal plane in the north (c.f. Fig 2).

The aquifer system consists of a series of Tertiary limestone formations containing eogenetic karst [25, 26, 27]. The term eogenetic refers to the early stage of karst porosity evolution at low burial, involving surface processes and meteoric diagenesis [28, 29]. The limestone formations include, from north to south and stratigraphically from top to bottom, the Aymamon limestone, the Aguada limestone, the Cibao Formation, and the Lares limestone (Fig 2).

The Tertiary limestone units are locally covered with Quaternary soils such as clay, blanket sand deposits, marine terrain deposits and stream valley alluvium [30, 31]. In the area of Vega Alta, where the present study was carried out, the aquifer system does not contain the Lares limestone formation. Due to the high annual rainfall and the warm climate of the area (average annual temperature about 25°C), the limestone units and soils are highly prone to erosional processes, and climatic conditions are favorable for karst development. The karst conduit systems typically occupy only a small portion of the total aquifer porosity [9]. Nevertheless, the karst conduit system may have a major impact on the hydraulic behavior of the total karst system. The frequency of sinkholes is < 1% in most locations and is much less in Vega Alta than in other municipalities of the north coast. As a result, point recharge into the aquifer is minor, leading to relatively low groundwater level fluctuations [32].

The limestone formations in northern Puerto Rico are slightly or moderately karstified [33]. Generally, the hydraulic conductivities of these units decrease exponentially from a maximum of about 2,040 m/day in the upper Aymamon limestone to a minimum of about 0.04 m/day in the basal Lares limestone [32]. In Vega Alta area, a first set of horizontal hydraulic conductivities (K values) and their vertical distribution over the model layers were defined by Sepúlveda [34] based on slug tests data conducted in 23 multiport wells with different monitoring ports at various depths. The slug tests indicated that K values ranged from 0.06 to 305 m/d, with generally higher values near the water table. The karst upland plateau in Vega Alta encompasses numerous closely spaced deep sinkholes and few gentle depressions in Aguada limestone and Aymamon limestone, respectively. Hydrological investigations [32, 35] demonstrate highly variable porosity and high permeability contrasts in short distances reflecting the variable distribution of conduit porosity in the north coast and suggest that water-bearing conduits are present between Rio Camuy and the Aguadilla area.

**Fig 3:**
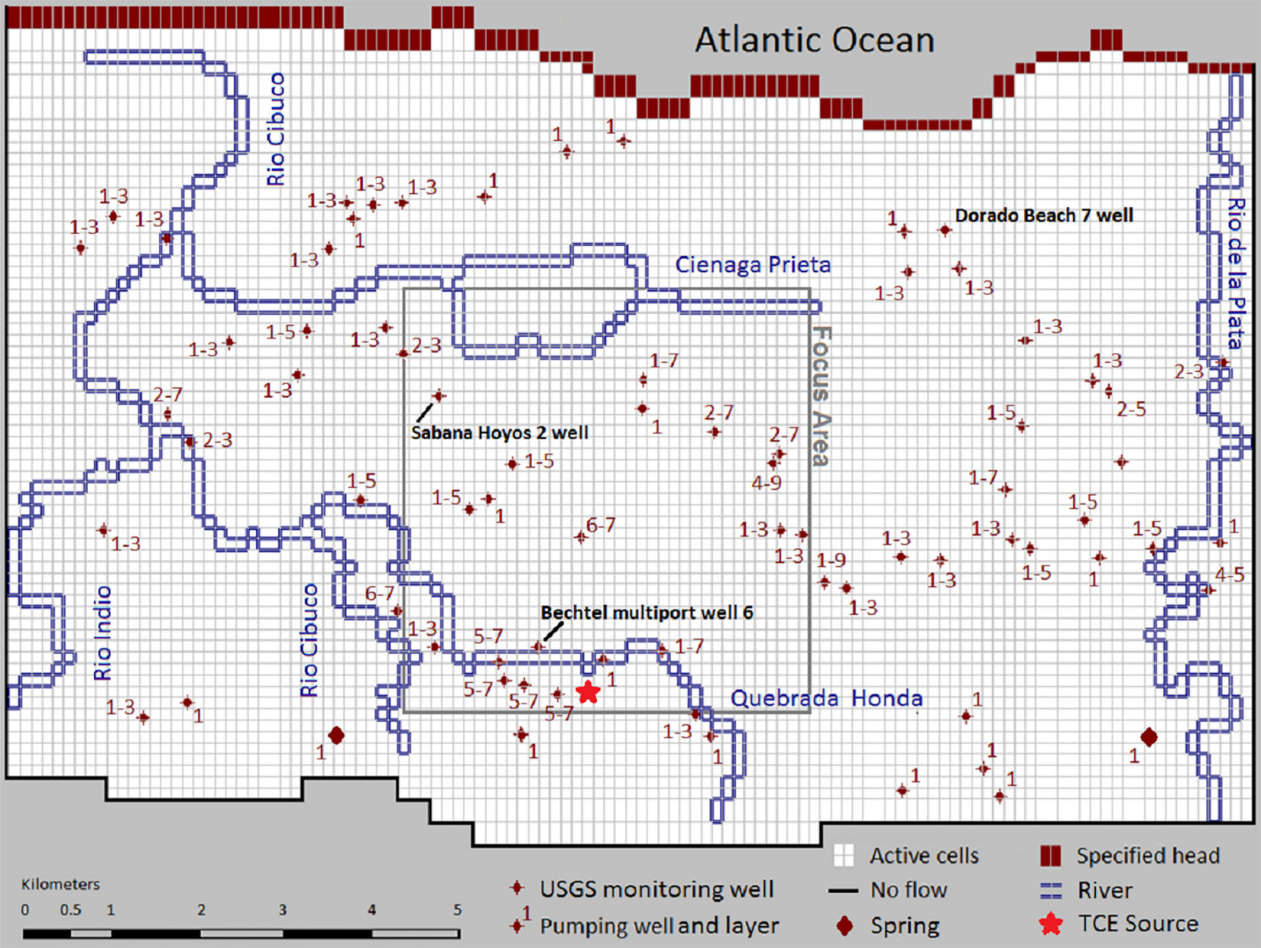
The model domain and defined boundaries. The domain extends beyond the two rivers and consists of focus area, location of TCE source, springs, USGS monitoring wells, and groundwater extraction wells. The well labels indicate the range of layers from which the groundwater was extracted.

The presence of caverns in test wells drilled in Hatillo and Isabela in the north and northwest of Puerto Rico has been verified by Rodriguez and Hartley [36]. Using aerial photographs and geophysical methods, Rodriguez and Richards [37] investigated a few sites in northwestern Puerto Rico to detect the presence of conduits and probability of conduit-controlled groundwater flow based on presence of natural potential anomalies. The exact locations of highly transmissive features such as cavities, conduits and fractures in central northern aquifers, however, are uncertain; and no dye tracer or cave diving evidence exists for locations and properties of subsurface conduits discharging groundwater into the northern coastal plain or the submarine outcrop of the aquifer [38]. Rodriguez [39] classified the springs in the north coast limestone based on their hydrodynamics and suggested that some springs are interconnected by an integrated conduit network. Mackovic spring (with limited discharge data) in Vega Alta exhibits little short-term response to rainfall, while Maguayo spring (with flow range of 147 to 2,789 m3/d) in Dorado exhibits a strong short-term response to rainfall and therefore they are known as diffuse-flow and conduit-type minor springs, respectively (see Fig 3).

The rainfall in Puerto Rico varies geographically and seasonally, with February being the driest month and September and October the wettest months [40]. There is a dry period starting in December and usually ending in March or April, followed with a spring rainfall period in April and May, an erratic, semidry period in June and July, and a wet season from August through November [33]. The average annual rainfall in the area of the North Coast limestone aquifer system is about 180 cm (NOAA). With a tropical climate and a high average level of solar radiation, the area experiences high evapotranspiration and receives relatively low rainfall intensity and duration, especially in winters. The average annual evapotranspiration is about 110 cm, or 63% of the average annual rainfall [33]. In Vega Alta area, assuming a minimal runoff, a preliminary estimate of annual aquifer recharge rates for watersheds in Puerto Rico was obtained by Giusti [33] from a correlation of evapotranspiration vs. rainfall (up to 84 cm recharge for a mean annual precipitation of 200 cm). Gómez and Torres-Sierra [41] estimated the spatial distribution of recharge for Vega Alta aquifer and developed a 2-dimensional groundwater model to assess the impact of additional withdrawals on groundwater balance.

#### Contamination concerns

The focus area of the present study was the unconfined Vega Alta limestone aquifer, which is a part of regional North Coast limestone aquifer. The aquifer consists of Aymamon and Aguada limestone (the two top layers in Fig 2) and is underlain by Cibao Formation in the south and saltwater-freshwater interface in the center and to the north. It is located in the Vega Alta and Dorado municipalities in the central part of the north coast of Puerto Rico between the municipalities Vega Baja and Toa Baja. The study area is bounded by the USGS Vega Alta quadrangle and the low permeability Cibao Formation to the south, the Atlantic Ocean coast to the north, the Rio de la Plata River to the west, and the Rio Indio River to the east (Fig 1).

Because of the high aquifer productivity, among other reasons, many pharmaceutical, chemical, and manufacturing industries have settled in the North Coast of Puerto Rico, with subsequent growth in population. Many of these industries rely on the use of hazardous materials, which can enter the karst groundwater from accidental spills and deliberate disposals. Many sinkhole depressions have also been used as clandestine waste disposal sites. Water quality surveys in Puerto Rico have shown a vast contamination of the North Coast limestone aquifer [42]. Contamination with chlorinated chemicals, which have been measured in a large percentage of sample wells, is of great concern. Extensive contamination has resulted in the closure of 41% of drinking water supply wells by 1987 [43]. Since then, there have been more closures, and recently, groundwater has been avoided as a primary source of public water supply in northern Puerto Rico [23].

Up to 49 thousand cubic meters per day of groundwater was extracted in 1991 from 20 public supply wells in Vega Alta and Dorado for public water supply and private usage (industrial, commercial, and agricultural) [40]. Being among the most productive aquifers of the island, this aquifer is at the same time one of the most contaminated. Volatile Organic Compounds (VOCs) used in the metal, electronic, and dry cleaning industries have been leaching from beneath an Industrial Park (see Fig 1) for decades. It was first considered contaminated in 1983 when water samples from 17 of 90 wells collected at the Vega Alta Public Supply Wells site (Latitude: 18.41806, Longitude: -66.33028) were identified to contain high concentrations of methylene chloride, extractable organic compounds, trichloroethylene (TCE) and other VOCs [44].

The US Environmental Protection Agency (EPA) was concerned about health effects of this contamination, and the contaminated wells were immediately shut down by the Puerto Rico Aqueduct and Sewer Authority (PRASA) being responsible for the operation and maintenance of the public water supply system in Vega Alta. In 1984, the site was included in the Superfund National Priorities List (NPL) as “Vega Alta Public Supply Wells” Superfund site. The Vega Alta landfill and the Industrial Park have been recognized as possible sources for TCE and other VOCs. The Vega Alta limestone aquifer was estimated to contain 5900 kg (in 1990) and 5800 kg (in 1992) of TCE, and solute TCE influx into the aquifer was estimated at 9.98 kg/yr under long term average net recharge rates [22]. The drinking water supply of about 40,000 people in Vega Alta municipality depends upon the health of the highly vulnerable karst aquifer. Establishing a thorough understanding of the hydrogeology of this major karst aquifer system is important for water resources and groundwater management and also provides the foundation for predicting the contamination migration.

### Model Setup and Calibration

To illustrate the application, performance, and limitations of the EPM modeling approach in evaluating the release and transport of contamination in karst aquifers, a MODFLOW model was developed and paired with MT3DMS; both were implemented in Visual MODFLOW Standard 2011.1 [45]. The variably-spaced grid is composed of 76 rows and 113 columns, encompassing a total area of 132.5 km^2^ (Fig 3). The grid is oriented south-north in order to align the model columns with the dominant coastward direction of groundwater flow. The rectangular cells have a maximum size (∆X×∆Y) of 236×248 m^2^ and a minimum size of 118×124 m^2^. The final grid consists of 8588 cells in each layer, with the finest refinement around the contamination source (the eastern side of the Industrial Park) and water extraction wells. The unconfined aquifer was subdivided into 12 layers with basis elevations of -3.05, -15.24, -22.86, -30.48, -38.1, -45.72, -53.34, -60.96, -68.58, -76.2, -86.87, and -97.54 m a.s.l, whereas the last elevation corresponds to the base of the karst aquifer (lower model boundary). The subdivision is based on similar hydraulic conductivities and initial TCE concentrations in these layers [46]. The saturation thickness of the top layer varies as a function of the water table.

Transient simulations are needed to analyze time dependent variables such as the response of water table in aquifer systems to transient signals like seasonal precipitation pulses. The transient flow simulations are run using monthly rainfall between February 1983 and December 2011 (29 years), and also using a refined daily rainfall between June 2004 and December 2005 (19 months) under the same conditions to evaluate the performance of the flow model in a greater temporal resolution.

The Atlantic Ocean coastline in the north is defined as a specified (constant) head boundary of the model with zero head at the sea-level. Predominantly south to north groundwater flow, suggested by historical water level data, implies no-flow boundary conditions along the western and eastern boundaries (c.f., Fig 3). These assumptions are consistent with previous modeling efforts [34, 41, 46] and are constant throughout the simulations. The unconfined aquifer bottom is underlain by the Cibao formation toward the south and by a static saltwater interface (groundwater with estimated dissolved solids exceeding 35,000 mg/L) toward the northern part of the area. In the Vega Alta karst system, the southern Cibao formation is almost impermeable with very low effective porosity due to the existence of clay layers and the absence of karst features. Therefore, a no-flow boundary for each layer is used as the southern and bottom boundary of the model, as well as the northern model boundary (Atlantic Ocean) in all layers except for the topmost two layers in the north.

Recharge is a key component of the hydrological balance in groundwater systems, and it is often difficult to quantify this parameter since it depends on variety of complex factors such as runoff and evapotranspiration. Due to the relatively low relief (maximum altitude of around 150 m a.s.l.) in the Vega Alta area, the average rainfall depends only little on the altitude (slightly greater values at higher elevations), which is confirmed by NOAA data. Therefore the model assumes that the rainfall is independent of the elevation, and rainfall data are only taken from Dorado rain gauge in the study area for model setup. Diffuse infiltration of rainfall through permeable outcrops is the major source of recharge to the aquifer. The net transient recharge is spatially distributed in six zones over the aquifer with rates calculated by multiplying the monthly rainfall to the calibrated discrete aerially-weighted conversion factors of 3%, 6%, 9%, 16%, 25%, and 31% of the net rainfall for the different geologic formations. The highest recharge rates correspond to the limestone outcrops and medium and lower recharge rates for areas with thick blanket deposits and coastal clay deposits, respectively. Aerial recharge was considered as specified flux (Neumann) boundary condition.

The major streams flowing through the study area are Rio de la Plata (the longest in the island) and Rio Cibuco with drainage areas of 227 and 519 square kilometers, respectively (c.f., Fig 1). At base flow conditions, river stages measured at the intersections with highway 2 correspond to 1.52 and 0.91 m a.s.l respectively. Both water level stages are assumed constant. The rivers were implemented as a transfer (Cauchy) boundary condition in the topmost layer of the model, with riverbed conductance values used for river cells ranging from 4.645 to 371.61 m^2^/day, according to the studies of Monroe [30] and Gómez and Torres-Sierra [41].

There were 72 private and public pumping wells in the Vega Alta study area. Historically, well closure was the major reason for the decline in groundwater withdrawals, and the lost water public supply was compensated from surface-water sources [47]. Withdrawal rates and filter depth data were taken from field measurement by Sepúlveda [34] and PRASA before 1992, and were assumed constant between 1992 and 2011. The transient model requires initial conditions for the hydraulic head and contaminant distribution. A map of water levels measured in February 1983 [46] is used to define initial conditions of the transient flow model. The effects of uncertainty in initial conditions on simulation results decrease as the simulation progresses. Therefore, simulations were given sufficient “spin-up” time before the period being modeled to limit errors associated with possibly erroneous initial conditions.

**Fig 4:**
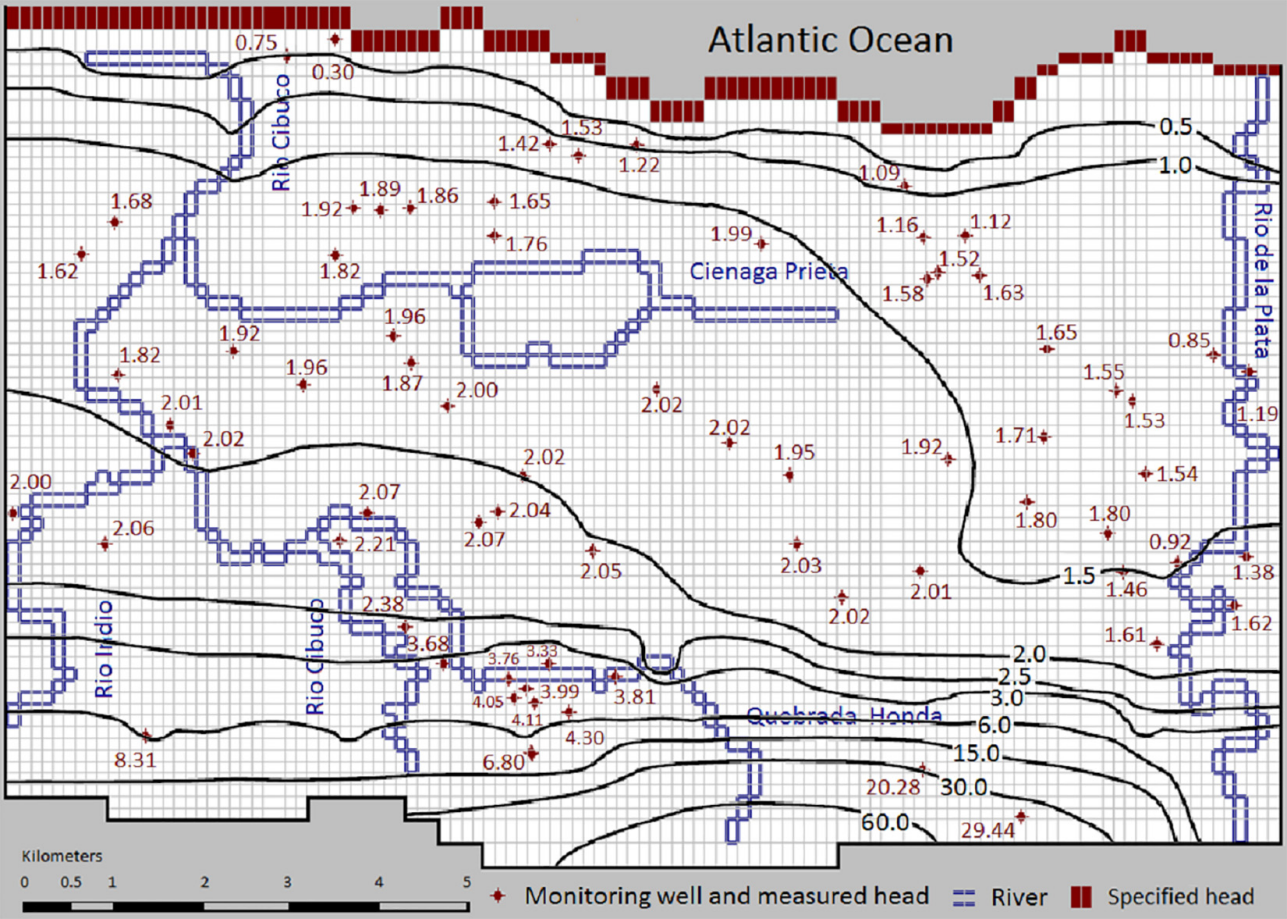
Calibrated steady-state water table contours for February 1983. The simulated groundwater levels were used as the initial conditions for the transient flow model. The monitoring well labels represent the measured groundwater heads (m above sea level).

Measured water table heads of February 1983 [48] in 70 wells were used for steady-state calibration to estimate the initial conditions for subsequent transient simulation. Parameters used for steady-state flow calibration are net recharge and hydraulic conductivity. These values are slightly adjusted and refined during the manual steady-state calibration processes to match modeled and spatially observed hydraulic heads (Fig 4).

**Fig 5:**
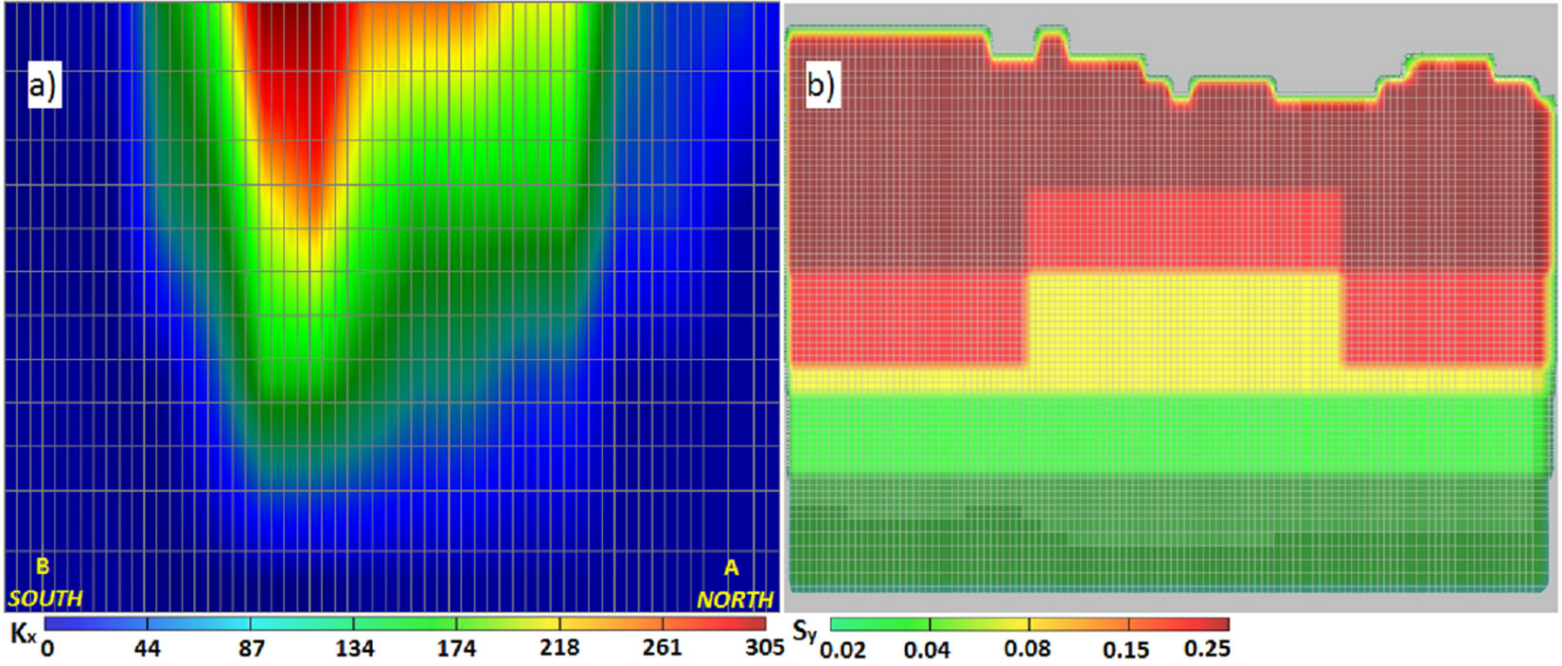
Variability of aquifer hydraulic parameters. (a) Vertical distribution of calibrated hydraulic conductivity (m/day) along cross section A-B (the plot is 50 times exaggerated in the vertical axis); and (b) specific yield of all model layers.

Transmissivity within the upper limestone aquifer is largely regulated by the thickness of the freshwater lens that thins landward and coastward [24]. The distribution of hydraulic conductivities first estimated by Sepúlveda [34] were further adjusted for each layer in this model (Fig 5A), with lower values in the Cibao formation and higher values in the Aguada and Aymamon formations. The highest K values in the middle of model domain correspond to the upland plateau characterized by outcrops and closely spaced sinkholes which are surrounded by low rolling hills.

The same model parameters (hydraulic conductivity and recharge) used for steady-state calibration were used for the transient calibration to adjust the storage parameters. In the north of the karst upland plateau, with alluvial aquifer and beach deposits, transient calibration resulted in a specific yield (S_y_) of 0.25. The limestone aquifers of the karst upland plateau were calibrated with a S_y_ of 0.08; to the north, west and east of the plateau, where it transitions to the alluvial aquifer, all limestone units were calibrated with S_y_ of 0.15. The south of the karst upland plateau with clay lenses was calibrated with S_y_ of 0.02 after a transition area with S_y_ of 0.04 (c.f., Fig 5B).

A relatively sharp interface between fresh and saline water was estimated for the freshwater lens with a depth to the interface about 40 times of height of freshwater and the saltwater zone can extend up to 7 km inland of the Atlantic Ocean coastline [24]. The thickest part of the freshwater lens (exceeding 100 m) generally lies along a line where the interface with an approximate slope of 2% intersects the base of the aquifer (i.e. southern Cibao Formation).

**Fig 6:**
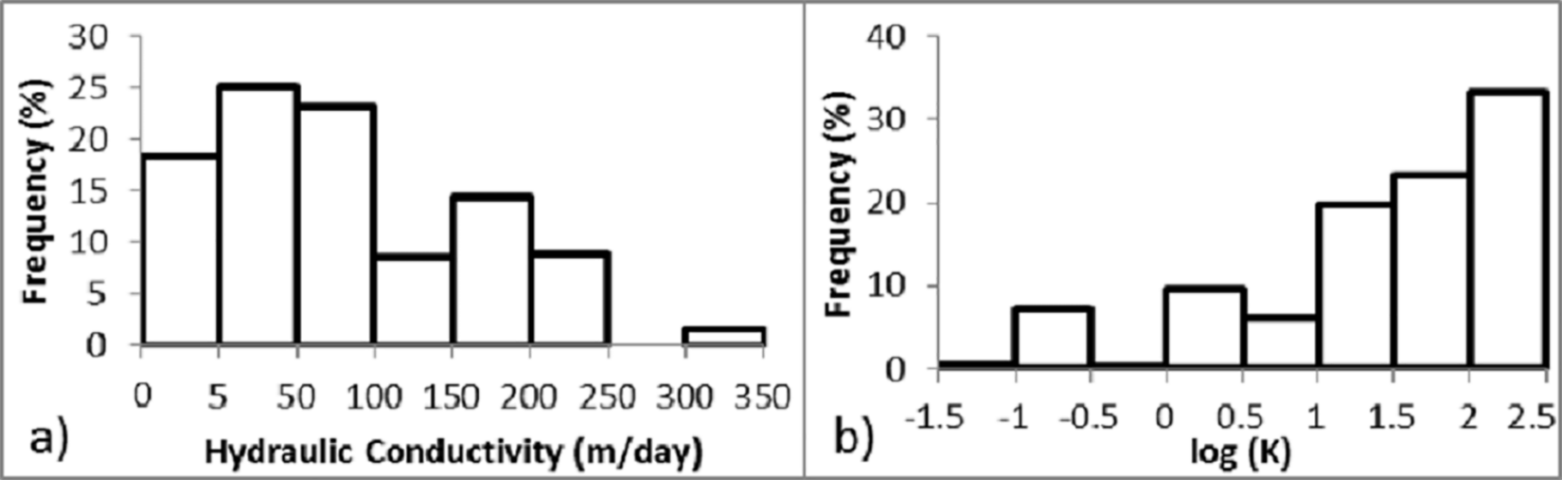
Frequency distribution of calibrated hydraulic conductivity. (a) Calibrated hydraulic conductivity is positively skewed; and (b) Logarithmic hydraulic conductivity log(K) is negatively skewed.

Commonly, the frequency distribution of the hydraulic conductivity is closer to log-normal than normal [49] in karst aquifers, but could be positively or negatively skewed [29]. The distribution of fracture aperture widths is generally assumed to follow a log-normal [50] or power-law distribution. Conduits spacing should therefore also follow a similar distribution, however, if present, a single conduit can dominate an aquifer [51]. The frequency distribution of the hydraulic conductivity of the present study is presented in Fig 6. Hydrogeological data sets often display a positive skewed frequency distribution, as hydraulic conductivity did in the present study (Fig 6A). The EPM favors a right-skewed distribution toward higher equivalent conductivity values, trying to approximate the individual highly-permeable pathways with an equivalent higher conductivity value in the model cells, which can be better seen in the logarithmic frequency distribution log(K) (Fig 6B). However, one should be aware that these plots do not represent actual field conditions with a possible conduit network, which can impose higher log(K) and potentially result in a log-normal distribution.

The transient transport model was run using monthly rainfall between January 1990 and January 2011. For the initial conditions, the transport model uses the head distribution in January 1990 computed by the flow model, and the TCE distribution in January 1990, the latter generated by linear interpolation of TCE concentration data measured in wells of the contaminated area [46]. Except for the TCE source cell near the eastern part of the Industrial Park (c.f., Fig 3) in the uppermost model layer, the TCE recharge concentration (C_RCH_) in the study area was set to zero. The TCE mass present as free phase in the source zone or adsorbed onto the media provides ongoing sources of dissolved contamination in the aqueous phase. The mixing of the dissolved TCE in the source zone is a function of the local groundwater flow field within the TCE entrapment zone. Although the influx concentration may vary depending on hydrological conditions, it is considered constant in the transport simulations. The subsequent development of the dissolved TCE plume is controlled by the regional groundwater flow field and various processes such as advection, dispersion, diffusion, adsorption, and a minimal decay.

Since chlorinated organic compounds tend to decay very slowly in groundwater, TCE was assumed conservative with a minimal first-order decay constant of 1.95 ×10^−6^ d^-1^ (a half-life of 975 years). A linear distribution coefficient (k_d_) of 0.2 l/kg (corresponding to a retardation factor of 3.95) was used in this study based on theoretical methods and field measurements on distribution coefficient for sorption of TCE in soil water systems with different organic carbon content [52].

Manual calibration of the transport model was performed based on comparing simulated and measured TCE concentrations between January 1990 and January 2008. Groundwater samples were collected quarterly from monitoring wells in the focus area by USGS and EPA. The environmental and hydrogeologic data were collected from recent studies and US Geological Survey [34, 46]. All data were compiled into a sustainable database in the Puerto Rico Testsite for Exploring Contamination Threats (PROTECT) Center in Northeastern University for current and future study.

### Model Evaluation

To compare the closeness of modeled values to measured values, the model’s estimation errors were calculated using two measures of root mean square error (RMSE) and mean absolute error (MAE). Also linear regression analysis was used to evaluate the dependency of water level fluctuations to precipitation events.

## Results and Discussion

One of the main objectives of the study is to evaluate the performance and applicability of the EPM approach to simulate flow and transport for a highly permeable karst aquifer. For this reason, efforts have been undertaken to challenge model results and thoroughly evaluate them against given data from the study site.

### Flow Model

**Fig 7:**
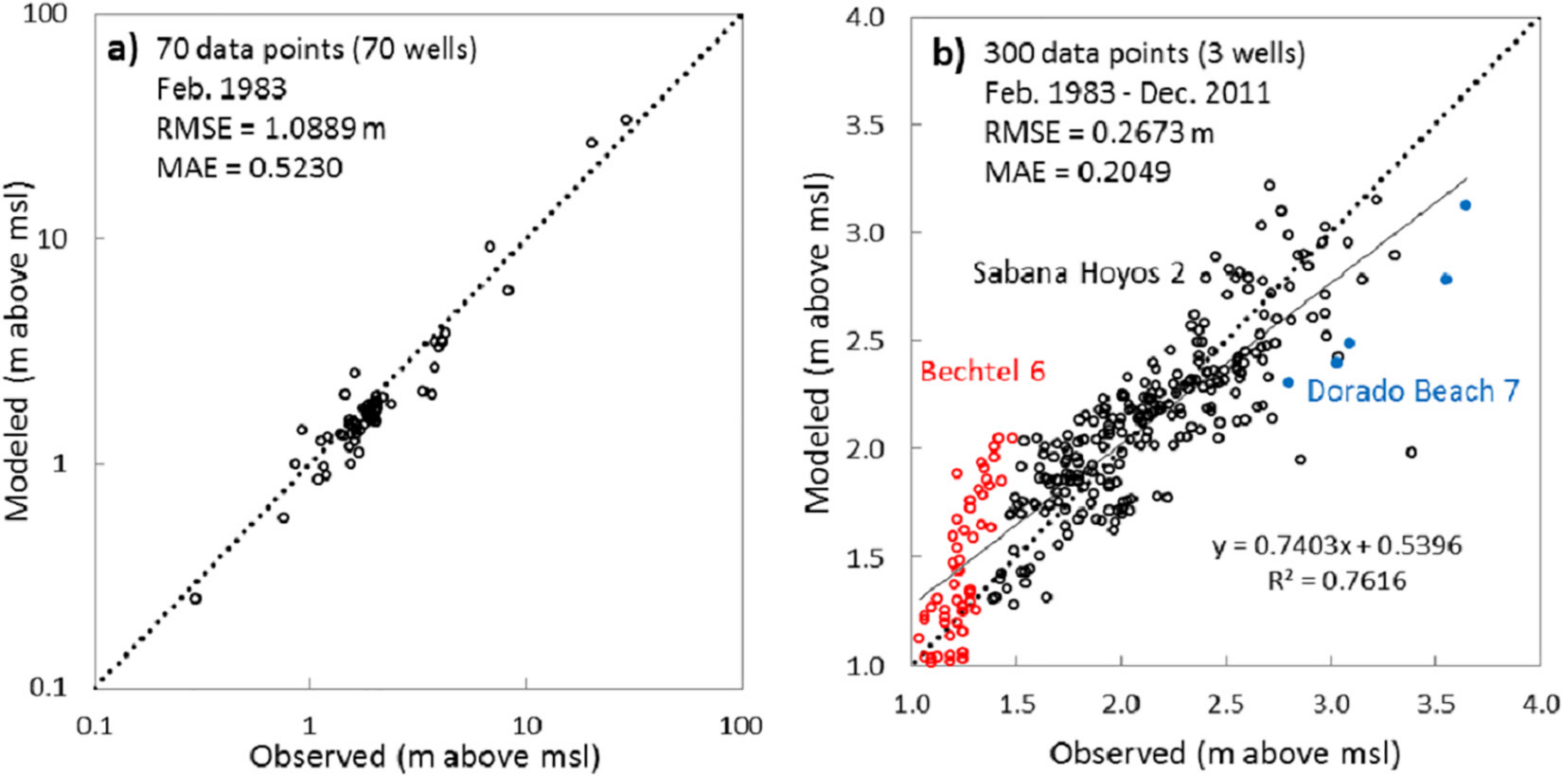
Scatter calibration diagrams of simulated versus observed hydraulic heads. (a) Steady-state calibration of the flow model (70 monitoring wells); and (b) Transient calibration of the flow model over three decades (3 monitoring wells).

The steady-state calibration of the groundwater flow model was accomplished based on minimizing the MAE and RMSE of the simulated potentiometric surface map (Fig 4). Using average conditions in February 1983, RMSE of the model is 1.0889 m and MAE is 0.5230 m for 70 steady-state calibration wells (Fig 7A), representing about 1.7% and 3.6% of the measured water level variations across this unconfined aquifer. The number of available measured heads for transient calibration were much fewer than steady-state data, and the only three water level stations used for transient model calibration (operated by USGS Caribbean Water Science Center) are Dorado Beach #7, and Sabana Hoyos #2 and Bechtel #6 multiport wells (see Fig 3). The long term transient calibration results are presented in Fig 7B as a scatter plot diagram. Based on a statistical analysis of transient model results, the calculated low errors of 0.2049 m (MAE) and 0.2673 m (RMSE) indicate satisfactory simulation of water table variations in those stations. The linear trend line of data (R^2^ = 0.6867) is slightly misaligned from the expected 45° line. A Pearson correlation coefficient (R) of 0.8186 between simulated and observed hydraulic heads indicates generally good temporal correlation between observed and simulated water tables. Taking into consideration the used equivalent porous media approach, these values suggest satisfying results for the model calibration. Higher values of hydraulic head are slightly underestimated and lower heads are slightly overestimated by the model. This suggests that the recharge in dry seasons is overestimated, while it is underestimated in wet seasons.

The simulated steady-state water balance in the aquifer demonstrates that the total aquifer recharge mostly originates from precipitation (98.69%) and river infiltration (1.31%). Groundwater flow in the study area discharges mostly into the Atlantic Ocean (19.62%) in the uppermost two layers, and extraction wells (74.41%). Except the two fourth order springs (together discharging 1.05%), no major springs have been identified in the Vega Alta area (Fig 3), and surface rivers drain only 4.92% of the total groundwater discharge. As net recharge rates increased (decreased) or pumping rate decreased (increased) during the transient simulations, discharge rates to the rivers and to the Atlantic Ocean increased (decreased). Due to absence of natural drainage features (such as wetlands or lagoons); in Vega Alta area more groundwater is available for withdrawals than in adjacent aquifers [53]. As a result, the impact of excessive pumping from this productive unconfined aquifer on water table elevations and flow patterns are significant. The head contours are locally deviated around highly productive wells and the steady-state flow is diverged eastward close to Rio de la Plata River (see Fig 4). During extreme rainfall events, the surface rivers discharge to aquifer because the hydraulic gradient towards the aquifer increases.

**Fig 8:**
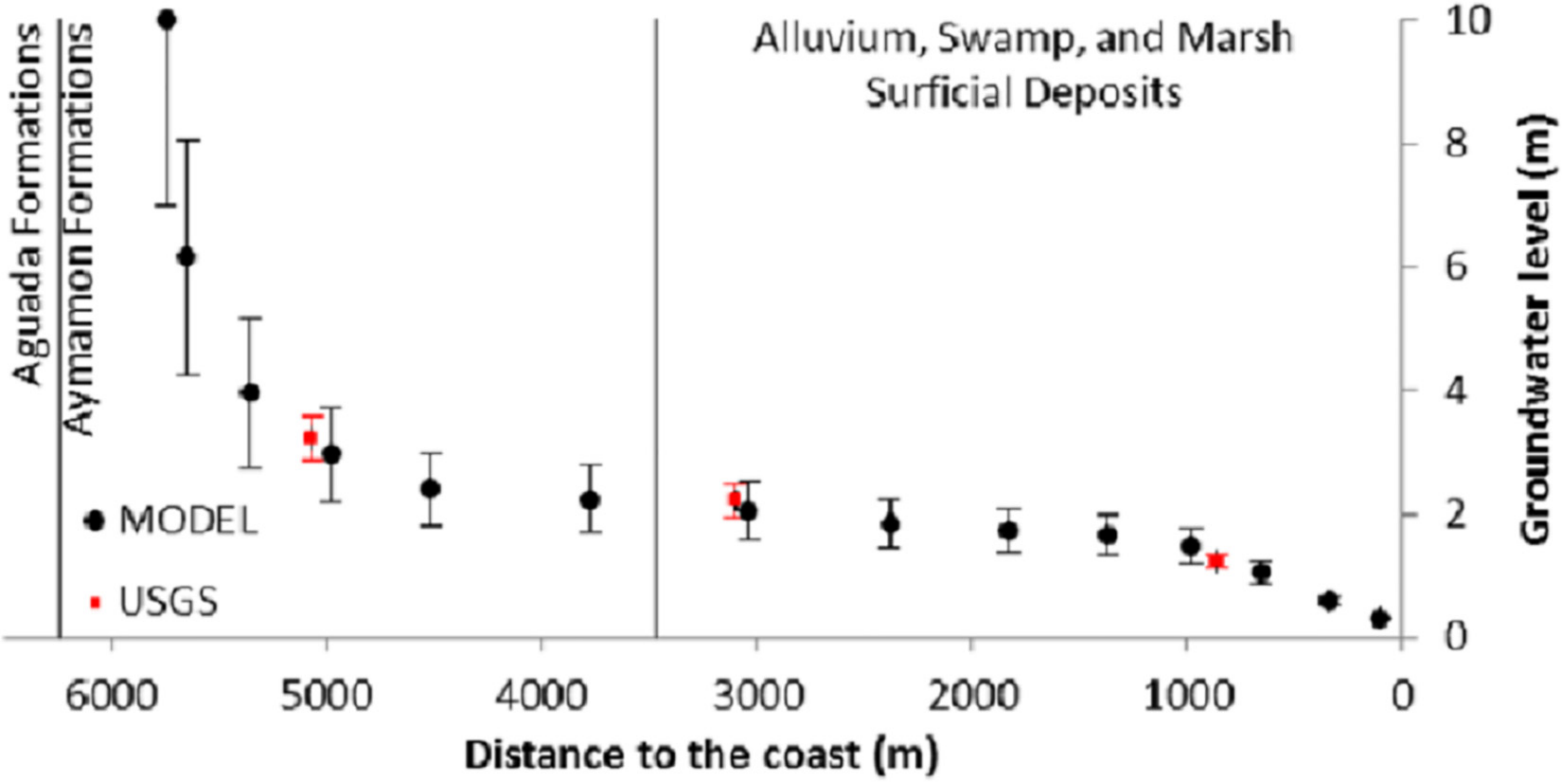
Simulated and measured groundwater levels vs. variable distance to coast. The mean and standard deviation for water level fluctuations in 3 USGS monitoring wells (red) and simulated points along the cross section A-B (see Fig 1). Distance dividers indicate the limit of surficial deposits and the Aguada-Aymamon intersection.

As the distance from the coast increases (>6700 m), the Aguada formation at the bottom of the aquifer becomes shallower and reduced in thickness. As a result, the depth-averaged storativity and conductivity values decrease and the average groundwater level rises faster relative to sea level (Fig 8). The magnitude of seasonal fluctuations depends upon the distance from the constant head boundary condition in the north. The low hydraulic gradient in the central part of the model reflects high hydraulic conductivities (c.f., Fig 5B), while slightly steeper hydraulic gradients reflect the relatively lower hydraulic conductivity in the northern model part. The steepest hydraulic gradient in south reflects the lowest values of hydraulic conductivity in the Vega Alta aquifer, which is a typical relationship between different zones in karst aquifers [5]. The water-table gradient is almost flat (0.57 to 0.76 meters per kilometer) in the Aymamon limestone. It increases to the south in the Aguada limestone (2.85 to 3.79 meters per kilometer) toward the outcrop areas of the Cibao formation (model boundary).

**Fig 9:**
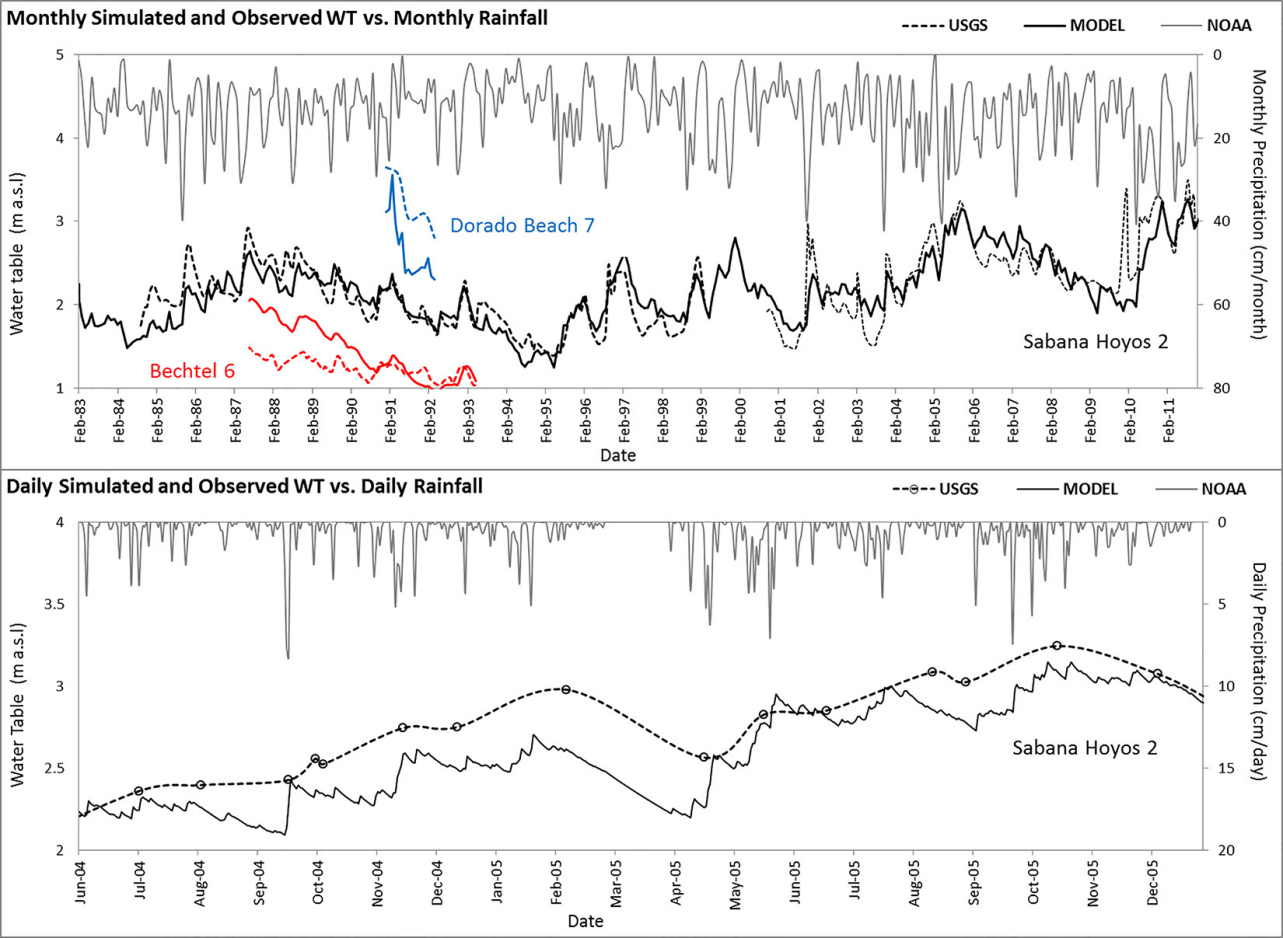
Monthly and daily averaged hydrogeologic conditions in Vega Ata aquifer. Simulated (solid line) and observed (dotted line) monthly water level hydrographs of three USGS wells vs. precipitation between 1983–2011 (upper); simulated and observed daily water levels in one well between June 2004 –December 2005 (lower).

Fig 9 shows the long term variation of the water table at three USGS wells and the water table rise of about 0.76 m at Sabana Hoyos #2 well over the last three decades. The rise is due to greater mean annual rainfall and the shutdown of some of the extraction. The simulated transient heads agree well with observed values, validating the assumption of constant withdrawal rates. This also suggests the reliability of EPM approach for replicating annual groundwater level fluctuations in northern Puerto Rico karst system, which agrees well with similar studies [5, 7, 12, 13] but provides validation in higher temporal resolutions (i.e. monthly and daily). Intermediate-term (several years) variation in water table corresponds to wet years, dry years and groundwater withdrawal activities. After 1967, the next severe dry period of the twentieth century in Puerto Rico was from 1993 to 1995 [54], when the annual rainfall accumulation was below normal with the greatest rainfall deficit.

Precipitation is often higher in winter than in summer. Therefore, the karst storage may not be fully recharged in summer and can demonstrate seasonal water table variations. The study results reveal a strong relationship between rainfall and water table fluctuations, demonstrating that the groundwater table was mostly recharged by the rainfall. In addition to climatic conditions, water table fluctuation also responds to the closing of contaminated public water supply wells and the operation of new ones between February 1983 and April 1992, after which there have not been more wells reported as closed. The results illustrate that the water table level achieved its overall minimum in 1995 and maximum in 2011.

Monthly rainfall intensities varied between 0 and 42.67 cm/month, with an average intensity of about 14.73 cm/month. The water table responds soon after the occurrence of intense rainfalls which suggests rapid infiltration and limited delayed recharge through vadose zone. The match between the observed and simulated response of the water table to rainfall events can be well reproduced by the model (Fig 9). For example, short-term seasonal groundwater levels in 2005 varied up to a maximum of 0.6 m according to both measured and simulated data (Fig 9). Analyses of different recharge events suggest that aquifer replenishment in the model responds greatly to rainfalls of any magnitude. It can be observed in the model results that the increasing response to short-term precipitation events, such as storms, is faster than the groundwater decline following the storm.

**Fig 10:**
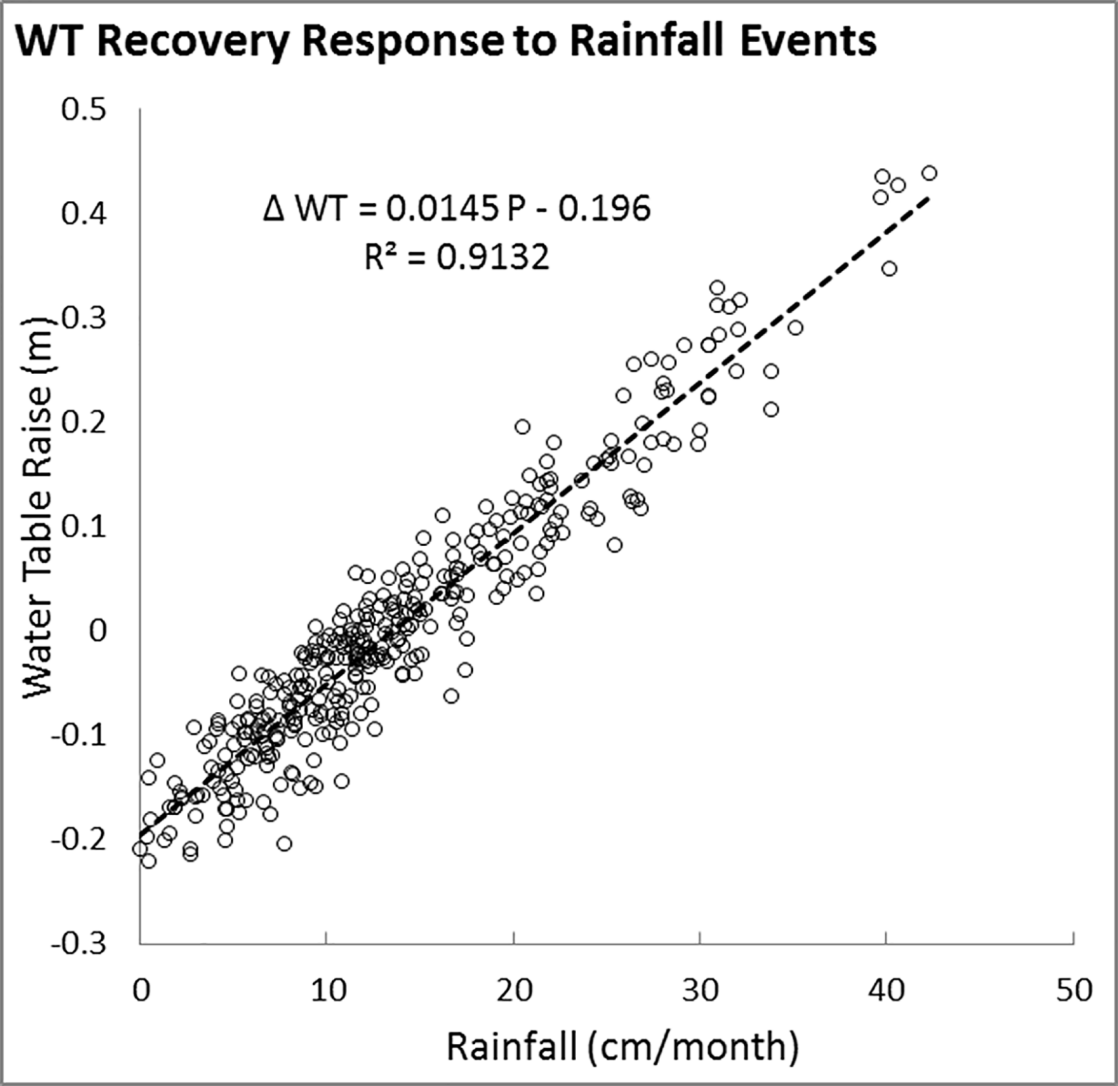
Scatter diagram of simulated water level recovery as a linear function of rainfall. On average, a monthly rainfall less (more) than 13.5 cm would lower (increase) the water table in USGS Sabana Hoyos #2 well.

Water table recovery, which is important in aquifer protection and sustainable water resource management, is affected by factors including precipitation rate, recharge and discharge rates, porosity, topography, and geologic structure. The water table recovery after each precipitation event was faster in the wet season and is observed several hours or several days after rainfall events in the study area. The linear correlation of monthly rainfall intensities with the water table recovery observed by Chuanmao [55] is acknowledged by the model results of the present study in the Vega Alta aquifer. It is thus possible to estimate the water table raise in each month based on monthly rainfall intensity. For example, the linear regression equation for water table recovery at Sabana Hoyos #2 well (R^2^ = 0.9132) is shown on Fig 10, where ∆*WT* is monthly recovery of water table (m), and *P* is the monthly precipitation (cm/month).

The hydrogeologic effects of subsurface conduit networks cannot be reproduced using an EPM modeling approach, nor can be well detected using limited number of head monitoring wells in this study. The developed flow model for Vega Alta karst aquifer adequately simulates the spatial distribution and high-resolution temporal variation of groundwater levels over 21 years. It also uses monthly-refined rainfall, and captures the water table fluctuations in a larger temporal resolution for a shorter time period using daily-refined rainfall. Other flow modeling studies in northern Puerto Rico (e.g. Sepúlveda, 1996; Cherry, 2001) only demonstrated EPM’s ability to regenerate steady-state regional hydrogeology, or transient simulations using very coarse rainfall resolution (e.g. annual).

### Contaminant Transport Model

**Fig 11:**
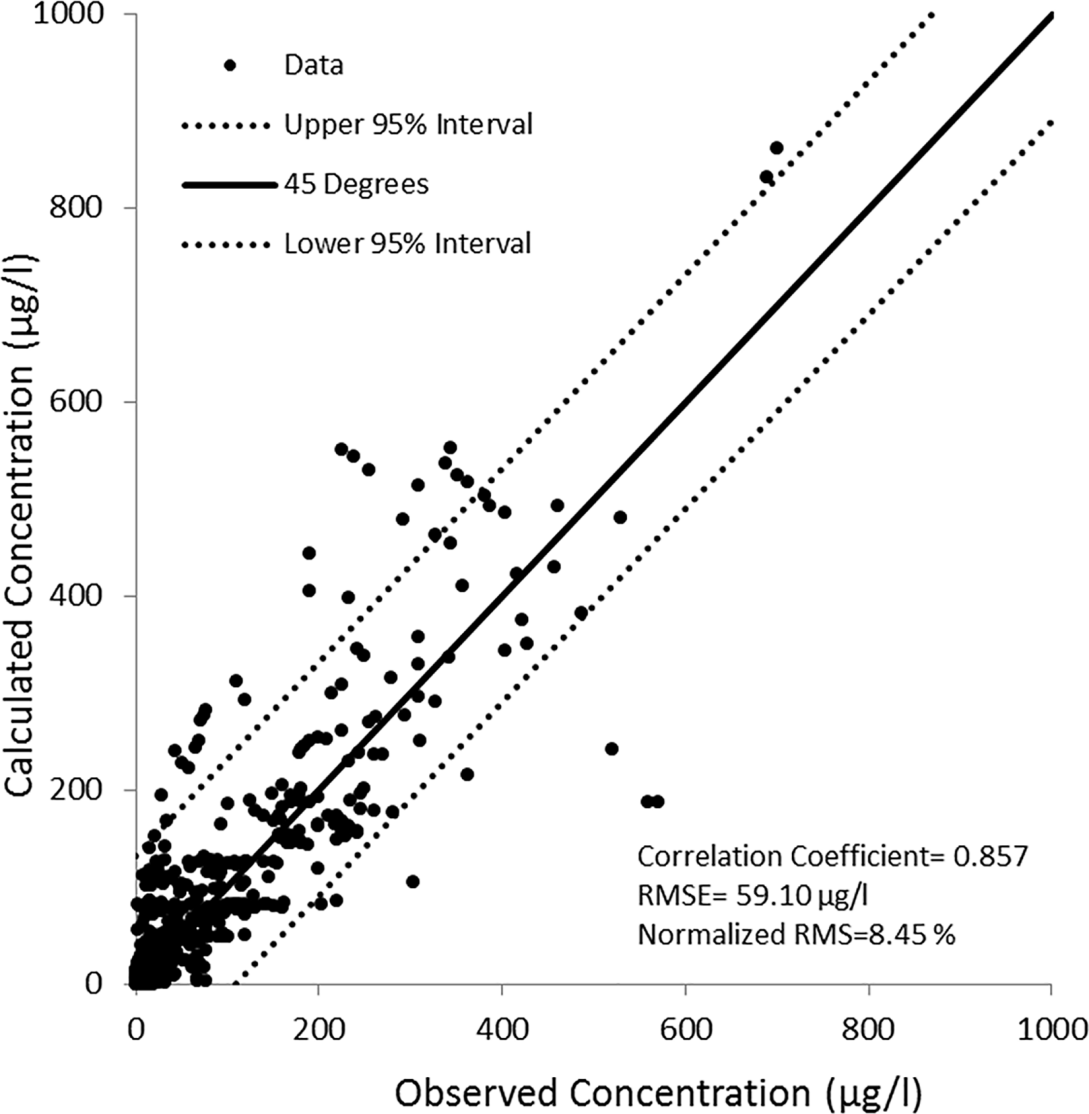
Transient calibration diagram for the transport model. Scatter diagram of simulated vs. observed TCE concentrations at various depths in 10 monitoring wells between January 1990 and January 2008.

The calibration was accomplished by adjusting C_RCH_ value to 1,700 μg/l in the TCE source cell. Based on this value, the average TCE influx from the source area can be calculated by multiplying C_RCH_ with the recharge rate and the TCE source cell size, resulting in 7.035 kg/yr TCE influx into the unconfined aquifer. To control the mixing of the TCE in the water, uniform scale-dependent longitudinal, transverse, and vertical dispersivity values of 3, 0.3, and 0.03 m, respectively, were resulted during the calibration for all model layers, with assuming a typical molecular diffusion coefficient k_m_ of 10^−5^ cm^2^/s. The calibrated model agrees well with observations in 10 monitoring wells in the area, and the correlation coefficient between simulated and measured TCE concentrations is 0.857 (Fig 11). Besides the limited field data used, the model simulation of concentrations produced an overall RMSE of 59.10 μg/l (normalized RMSE = 8.45%). This correlation suggests that the use of an EPM approach to simulate solute transport in an intermediate scale for this eogenetic karst limestone aquifer is justified. As noted by other studies [5], EPMs are not suitable for transport modeling in a local scale, where in-depth knowledge of distribution patterns of fracture and conduit networks is required to describe the complex transport processes.

**Fig 12:**
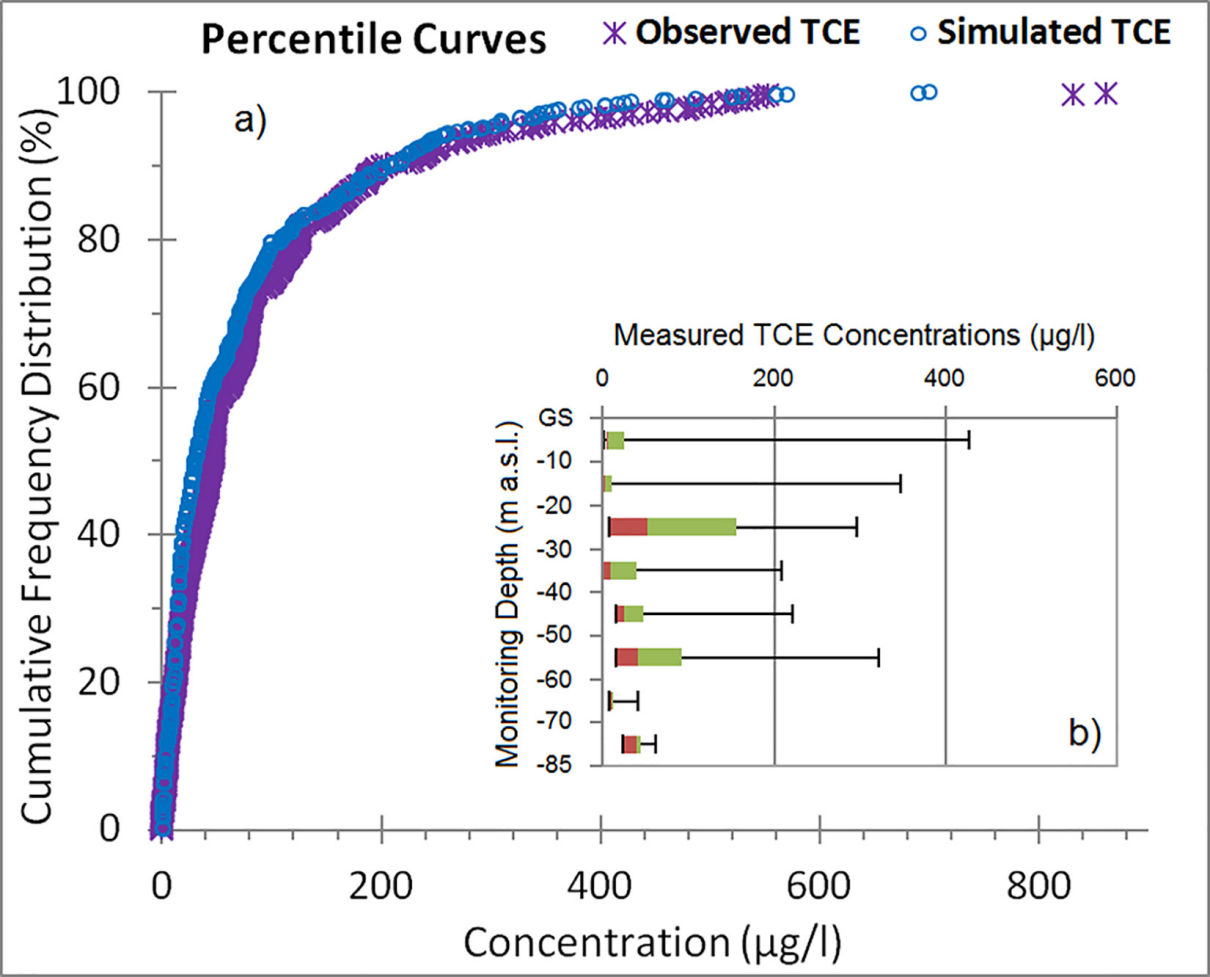
Cumulative frequency and vertical distribution of detected TCEs in groundwater. (a) Cumulative quantile plots of observed and simulated TCE concentrations were obtained from historical data and model simulations, respectively. (b) Box plots for the depth distribution of observed TCE concentrations indicate 5%, 25%, 50%, 75%, and 95% quantiles (GS: ground surface).

TCE concentrations were measured at several observation wells in discrete sampling zones at different depths with the deepest at -82.6 m a.s.l. Percentile curve for the simulated TCE concentrations show good agreement with that for the TCE measurements distributed over the focus area (Fig 12). High degrees of spatial and vertical variation in TCE concentrations indicate the necessity in three dimensional simulating of the complex karst environment. The zone of high TCE concentrations extends to the depth of the deepest wells in the area (-61 m a.s.l).

In northern Puerto Rico, 56.6% of samples had TCE concentrations above detection limit (DL) and 11.5% of those samples had TCE concentrations above maximum contamination level (MCL) of 5 μg/l established by EPA. The mean, median, standard deviation, and coefficients of variation (CV %) for all measured TCE concentrations were 83.7 μg/l, 46.3 μg/l, 112.6 μg/l, and 41.1%, respectively, indicating that concentrations were substantially skewed (γ: 2.78) toward lower values. The sampling frequency and distribution have decreased significantly over recent years causing spatial uncertainties [56, 57]. Historically no significant seasonal fluctuation of TCE concentrations was observed in the taken samples (Yu et al., 2015); therefore in 2007, EPA proposed to reduce the sampling frequency from quarterly to semi-annually or annually. No TCE concentration was reported for Mackovic Spring in Vega Alta or Maguayo Spring in Dorado, both located outside of the focus area. Variability of spatially detected concentrations is commonly associated with complex subsurface hydrodynamics that affect storage and mobility of contaminants.

**Fig 13:**
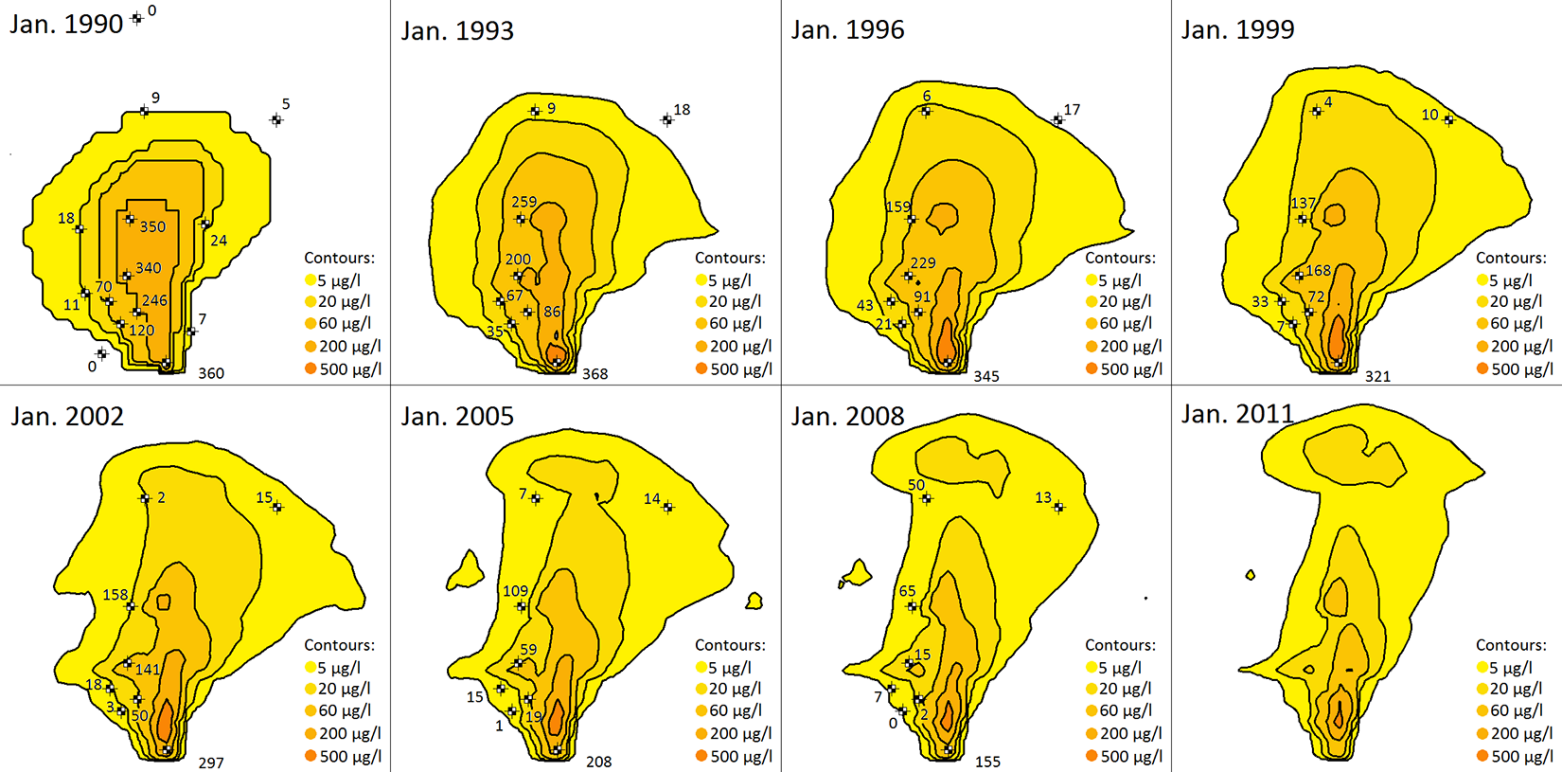
The migration and extent of TCE plume over two decades. Initial TCE contours were based on available data at January 1990, and simulated TCE contours and observed TCE data (unit: μg/l) were at eight monitoring wells at a depth of -30.48 m a.s.l between January 1993 and January 2011 in the focus area (4.6 × 4.8 km, see Fig 1). No observation data was available for 2011.

The initial distribution of TCE concentration and the simulated spreading of TCE plume in the focus area at a depth of -30.48 m a.s.l between 1993 and 2008 are presented in Fig 13. The presented observation data are interpolated values collected from monitoring zones between depths of -25.91 to -36.58 m a.s.l. The spreading of the plume is simply observed by the outermost concentration contour which corresponds to the MCL (5 μg/l). The dominant flow path in the focus area is slightly northeast and downward towards the deeper aquifer, and concentrations directly south of the Industrial Park are negligible.

Due to the very low transport velocities, the TCE plume is very unlikely to reach the Atlantic Ocean. Sensitivity analysis suggests that the plume would spread further downward and to the north if no sorption of TCE was accounted for. The vertical hydraulic gradient becomes smaller toward deeper parts of the aquifer and toward the Atlantic Ocean. Accordingly, the simulation indicates a slightly downward and eastward transport in addition to the general northward movement of the TCE plume especially in the shallower parts of the aquifer.

The closure of some public water supply wells on the right side of focus area until 1992 has pushed the slight northeast groundwater direction further to the east. The unknown preferential flow paths and complex network of fractures, conduits and channels in this karst aquifer causes irregular spreading patterns and affects the contaminant dispersal and storage mechanisms. Measured TCE concentrations in different wells within the focus area had different temporal patterns reflecting high subsurface anisotropy and heterogeneity and different attenuation rates (also see Yu et al., 2015). In some wells, high variations of TCE concentrations were observed between consecutive monitoring events that are not captured by the model, e.g. after the plume had progressed enough (2005), the TCE concentrations in one site north of the plume started to increase gradually over time which was not seen by the model. The highest TCE concentration occurs directly downstream of the source cell and as the distance to the source increases, generally the TCE concentration decreases.

There are notable decreasing trends in the values and areas of TCE contamination on the temporal scale, as supported by the recent measured data. The total mass of TCE in Vega Alta aquifer was estimated as 5889.4 kg (equivalent to TCE volume of 4.033 m^3^) in January 1990. Although the annual TCE influx (7.035 kg) was contributing to the subsurface plume, the total contamination reduced to estimated total mass of 2501.4 kg in January 2011. Besides sorption and natural attenuation, this is partially because public water supply wells (before closure) and industrial and agricultural pumping wells withdrew the aquifer within the extent of TCE plume in the focus area. The TCE removal rate is found to be higher at shallower layers where vertical hydraulic gradient is larger.

Freshwater resources within the northern karst systems of Puerto Rico are highly vulnerable to contamination. Especially the recent industrial and urban development and increased number of contaminated sites within the island lead to unintended contamination of the groundwater resources. The subsequent long-term contamination indicates a large capacity of the karst aquifers for storing and releasing contaminants which reflects potential lasting exposures.

## Conclusions

This paper evaluates the application and adequacy of EPM approach to simulate groundwater flow and contaminant fate and transport processes within highly permeable karst environments, which is crucial for managing water resources management purposes. Where little to nothing is known about the occurrence and properties of conduit and fracture networks, approximating the complex nature of karst systems with equivalent hydraulic properties may sufficiently reproduce the aquifer behavior at regional and intermediate scales. Locating those networks and identifying the associated hydrogeological parameters are impossible without large scale exploration efforts. Using limited field data, the developed MODFLOW and MT3D models in this study adequately simulated the spatial distribution and high-resolution temporal variation of groundwater levels and the decreasing trend of TCE plume over 21 years, respectively, for Vega Alta karst aquifer. Withdrawals from a large well field, as the main regional discharge feature, are evaluated to significantly impact the water table dynamics and the dominant direction of plume migration. The occurrence of preferential flow influences the flow conditions and leads to irregular spreading of TCE plume in the aquifer. The main limitations of equivalent porous media approach in karst aquifers are failing to capture groundwater hydrodynamics on a local scale, and to simulate transient turbulent flow through conduit network and its interaction with the matrix.
